# Longitudinal Associations between Food Parenting Practices and Dietary Intake in Children: The Feel4Diabetes Study

**DOI:** 10.3390/nu13041298

**Published:** 2021-04-14

**Authors:** Paloma Flores-Barrantes, Iris Iglesia, Greet Cardon, Ruben Willems, Peter Schwarz, Patrick Timpel, Jemina Kivelä, Katja Wikström, Violeta Iotova, Tsvetalina Tankova, Natalya Usheva, Imre Rurik, Emese Antal, Stavros Liatis, Konstantinos Makrilakis, Eva Karaglani, Yannis Manios, Luis A. Moreno, Esther M. González-Gil, on behalf of the Feel4Diabetes-Study Group

**Affiliations:** 1 Growth, Exercise, Nutrition, and Development (GENUD) Research Group, University of Zaragoza, 50009 Zaragoza, Spain; pfloba@unizar.es (P.F.-B.); lmoreno@unizar.es (L.A.M); esthergg@ugr.es (E.M.G.-G.); 2 CIBER Fisiopatología de la Obesidad y Nutrición, Instituto de Salud Carlos III, 28040 Madrid, Spain; 3 Instituto Agroalimentario de Aragón (IA2), Instituto De Investigación Sanitaria Aragón (IIS Aragón), 50013 Zaragoza, Spain; 4 Red de Salud Materno Infantil y del Desarrollo (SAMID), Instituto de Salud Carlos III, 28029 Madrid, Spain; 5 Department of Movement and Sports Sciences, Ghent University, 9000 Ghent, Belgium; greet.cardon@ugent.be; 6 Department of Public Health and Primary Care, Ghent University, 9000 Ghent, Belgium; ruben.willems@ugent.be; 7 Department of Prevention and Care of Diabetes, Technical University Dresden, 01069 Dresden, Germany; Peter.Schwarz@uniklinikum-dresden.de (P.S.); patrick.timpel@tu-dresden.de (P.T.); 8 Population Health Unit, Finnish Institute for Health and Welfare, 00300 Helsinki, Finland; jemina.kivela@thl.fi (J.K.); katja.wikstrom@thl.fi (K.W.); 9 Department of Pediatrics, Medical University of Varna, 9002 Varna, Bulgaria; iotova_v@yahoo.com; 10 Clinical Center of Endocrinology and Gerontology, Medical University of Sofia, 1431 Sofia, Bulgaria; tankova@iname.com; 11 Department of Social Medicine and Health Care Organization, Medical University of Varna, 9002 Varna, Bulgaria; nataly_usheva@hotmail.com; 12 Department of Family and Occupational Medicine, University of Debrecen, 4032 Debrecen, Hungary; rurik.imre@sph.unideb.hu; 13 Hungarian Society of Nutrition, 1088 Budapest, Hungary; diet.emese.antal@gmail.com; 14 National and Kapodistrian University of Athens Medical School, 11527 Athens, Greece; sliatis@uoa.gr (S.L.); kmakrila@med.uoa.gr (K.M.); 15 Department of Nutrition and Dietetics, School of Health Sciences & Education, Harokopio University, 17671 Athens, Greece; ekaragl@hua.gr (E.K.); manios@hua.gr (Y.M.); 16 Institute of Agri-Food and Life Sciences, Hellenic Mediterranean University Research Centre, 71410 Heraklion, Greece; 17 Department of Biochemistry and Molecular Biology II, Instituto de Nutrición y Tecnología de los Alimentos, Center of Biomedical Research (CIBM), Universidad de Granada, 18010 Granada, Spain

**Keywords:** prospective, home food availability, parental modelling, use of food as reward, permissiveness, European children

## Abstract

Food parenting practices (FPPs) have an important role in shaping children’s dietary behaviors. This study aimed to investigate cross-sectional and longitudinal associations over a two-year follow-up between FPP and dietary intake and compliance with current recommendations in 6- to 11-year-old European children. A total of 2967 parent-child dyads from the Feel4Diabetes study, a randomized controlled trial of a school and community-based intervention, (50.4% girls and 93.5% mothers) were included. FPPs assessed were: (1) home food availability; (2) parental role modeling of fruit intake; (3) permissiveness; (4) using food as a reward. Children’s dietary intake was assessed through a parent-reported food frequency questionnaire. In regression analyses, the strongest cross-sectional associations were observed between home availability of 100% fruit juice and corresponding intake (β = 0.492 in girls and β = 0.506 in boys, *p* < 0.001), and between parental role modeling of fruit intake and children’s fruit intake (β = 0.431 in girls and β = 0.448 in boys, *p* < 0.001). In multilevel logistic regression models, results indicated that improvements in positive FPPs over time were mainly associated with higher odds of compliance with healthy food recommendations, whereas a decrease in negative FPP over time was associated with higher odds of complying with energy-dense/nutrient-poor food recommendations. Improving FPPs could be an effective way to improve children’s dietary intake.

## 1. Introduction

Youth overweight and obesity prevalence has risen in the last decades, amounting to more than 124 million children and adolescents, of which 6% were girls and 8% were boys in 2016 [1]. This condition has been associated with several cardio-metabolic risks and diseases [2,3], which could track into adulthood [4]. Among the obesity-related factors, dietary behavior is one of the most relevant due to its strong relationship with energy balance [5].

Traditionally, the family is an important social context where children learn and adopt eating behaviors [6]. Especially during the first years of life, children’s learning about food and eating plays an important role in shaping subsequent food choices, diet quality, and weight status [7]. Consequently, children are strongly influenced by their parents, not only genetically but also due to the use of food parenting practices (FPPs) as parents are providers, models, and regulators of their children’s dietary intake and home food environment [8].

Children’s intake of specific food items is further limited by the home availability of those food items. Availability is one of the main parental practices that shape children’s eating habits as repeated exposure to food items enhances preference development [9]. Parents are the food providers for their children, and their preferences and selections have an impact on their children’s dietary habits, which suggests that the healthiness of the familiar home food environment is triggered by the parents [10].

Children’s intake of specific food items, such as fruits, vegetables, or milk, has been associated with the observation of their parents consuming these foods [11]. Similarly, it has been observed that unhealthy eating patterns of parents are associated with a similar pattern in their children [12].

Parents are also regulators of the food items their children consume. Specific food items might be used as a reward, whereas others might be restricted. It has been observed that excessive restriction of specific food items (e.g., energy-dense foods, sugar-sweetened beverages) may have an undesired effect, increasing the preference for them when they are available [13,14]. On the other hand, using food as a reward has been associated with unhealthy eating among children [6] because the food chosen as a reward is often unhealthy. It is worth noting that the foods used as rewards could become more attractive to children as they could see them as valuable [15].

Several studies have evaluated the predictive value of the previously mentioned FPPs [16,17] and other FPPs, such as emotional feeding, instrumental feeding [16], food involvement, and family dinner frequency [18] on children’s dietary intake. Longitudinal studies have mainly focused on changes in food availability at home [19,20,21] but not on practices such as role modeling of fruit intake, permissiveness, or using food as a reward. Moreover, to the best of our knowledge, studies assessing the relationship between changes in FPPs and their impact on children’s diet in the European context over time are not available.

Thus, this study aimed to investigate cross-sectional and longitudinal associations between FPPs and children’s dietary intake and to determine if changes in FPPs over time are associated with compliance with food intake recommendations in a large sample of European children.

## 2. Materials and Methods

### 2.1. Study Design

Healthy Lifestyle FOR Diabetes prevention (Feel4Diabetes study) is an intervention study conducted between 2015 and 2019 in six European countries: representing high-income countries (Belgium and Finland), low-income countries (Bulgaria and Hungary), and countries under austerity measures following the economic crisis (Greece and Spain). The Feel4Diabetes study aimed to develop, implement, and evaluate an evidence-based, cost-effective program to prevent type 2 diabetes mellitus (T2DM) across Europe, especially focusing on families from vulnerable groups. Thus, an intervention area and a control area in each country were defined. A detailed description of the Feel4Diabetes study has previously been published [22].

Children attending the first three grades of compulsory education and their parents, or main caregivers, were recruited through schools. In the Feel4Diabetes study, 11,396 families (children) were included, and caregivers were screened for T2DM risk using the FINnish Diabetes RIsk SCore (FINDRISC) questionnaire, which has shown to be a reliable tool [23]. Families were regarded as “high-risk” if at least one parent fulfilled the country-specific cut-off point for FINDRISC, which indicated increased T2DM risk. Parents identified as being at “high-risk” were invited to participate in the second-stage screening, which included a brief medical check-up. In the Feel4Diabetes-study, three measurements were performed in the final trimester of three consecutive academic years (2016–2018): baseline (T0), follow-up 1 (T1), and follow-up 2 (T2).

The Feel4Diabetes study adhered to the Declaration of Helsinki and the conventions of the Council of Europe on human rights and biomedicine and was approved by each local ethical committee (see the detail of this statement in the Institutional Review Board Statement section at the end of the document). Parents received an information letter in which they were informed about the purpose of the study and the process in which they were invited to be involved, while written informed consent was obtained from all participant parents.

### 2.2. Study Sample

Parent–child dyads with information reported by the same parent at both time points and with complete data regarding FPPs, parental education, gender, age, and self-reported weight and height, as well as children’s dietary intake, gender, age, and body mass index (BMI), were included in the analyses. Since some families included more than one child and shared the same reporting parent, in order not to duplicate parental information, we then randomly selected one child per family. This way, 800 children were removed from further analysis. Also, to exclude potential outliers and after testing the distribution of the sum of fruits and vegetables, children consuming more than seven servings (90 g/serving) per day of fruits and vegetables, considering fresh fruit, canned fruit, 100% fruit juice, and vegetables, were removed from the analyses (*n* = 255). Due to the longitudinal data assessment of the study and the fact that the availability of data from the same parent–child dyad was mandatory for inclusion, from the 11,396 families included in the “all families” group, 2967 (26.04%) parent–child dyads (50.4% girls and 93.5% mothers) had complete data for inclusion in this study.

### 2.3. Food Parenting Practices

A general questionnaire with questions on socioeconomic and health-related behaviors was delivered to the families and filled out by one of the parents at home. Four FPPs were included in the questionnaire, questions and answer options are shown in Supplementary Table S1:

- Home availability of three foods considered to be healthy: fresh fruit, fresh fruit juice, and vegetables, and five food items considered to be energy-dense/nutrient-poor: sugary juices, soft drinks, light soft drinks, sweets, and pastries and salty snacks.
- Parental role modeling of fruit intake: consumption of fruit in front of their children.
- Permissiveness: allowance of sweets and salty snacks whenever the child asks for them.
- Use of foods as a reward: defined as using sweets, salty snacks, or fast food as a reward for their children.

Intra-class coefficients (ICC) of test-retest showed good reliability for home availability of foods (ICC = 0.720 (0.625–0.794)) and for parental role modeling of fruit intake, permissiveness, and the use of food as reward ((ICC = 0.695 (0.563–0.793)) [24]. Responses for these questions were on a five-point Likert scale that ranged from “very often” (“always” for home food availability) to “never”. These categories were reordered to denote increasing use of the practice, from “never” to “very often” (“always” for home food availability). Questions, response options, and analytic coding of these variables for the analyses are explained in Supplementary Table S1. For better interpretability of results, home availability of nutrient-dense foods and parental role modeling of fruit intake were grouped as positive FPPs, whereas home availability of unhealthy foods, permissiveness of sweets and salty snacks, and using food as a reward were grouped as negative FPPs.

### 2.4. Dietary Intake

Food and beverage intake of the child was reported by the parent and measured using the question: “how often do you and your child usually consume the following foods and drinks?”, which they could answer by choosing one of the following eight options: on a weekly (less than 1, 1–2, 3–4, or 5–6 times per week) or daily basis (1–2, 3–4, 5–6, and more than 6 times per day). Beverages assessed for children were water (one glass or one cup), fruit juices (freshly squeezed or prepacked without sugar), soft drinks and fruit juices containing sugar, and soft drinks without sugar. Foods assessed were fruits and berries (fresh or frozen), fruits and berries (canned), vegetables, sweets, and salty snacks and fast food (ICC = 0.633, (0.371–0.822) [24]). To facilitate the interpretation of results, food items were grouped as nutrient-dense (fresh fruits, canned fruit, 100% fruit juice, and vegetables) and energy-dense/nutrient-poor foods (soft drinks and sugary juices, light soft drinks, sweets, and salty snacks).

### 2.5. Dietary Recommendations

For multilevel regression analyses, a dichotomous variable for each food item was computed to establish whether the child complied with dietary intake recommendations. This study used recommendations for dietary intake from the Irish Food Pyramid [25] since it includes recommendations as servings per day and specific cut-off points, suitable for use in children. Regarding water intake, although the Irish Pyramid recommends eight or more cups per day, we used the highest food intake category as recommended, which was equivalent to six servings per day. Also, to compute the total daily amount of fruits and vegetables consumed, two variables were calculated. The first, (F and V^1^), included fresh or frozen fruit and berries, fresh fruit juices, and vegetables, and the second, (F and V^2^), included the same food items as F and V^1^ plus canned fruits and berries. The cut-off point for compliance with recommendations was set at five servings per day for both variables. Nevertheless, the recommendations for each item included as fruit and vegetables, e.g., fresh fruit, or vegetables, was set at 1–2 times per day. For energy-dense/nutrient-poor foods, e.g., soft drinks, the Irish Food Pyramid determines no recommended number of servings per day, since they are not needed for good health. However, in order to establish a cut-off point, ideal intake of these foods was considered as once or less per week.

### 2.6. Anthropometric Measurements

Children were measured following standard procedures by trained researchers [26]. Bodyweight was measured in light indoor clothes and bare feet with a calibrated scale (Type SECA 813). Body height was measured with a wall stadiometer (Type SECA 217). Both measurements were performed twice, and a third assessment was carried out if the difference between the two measurements was greater than 0.1 kg or cm, respectively. Children’s BMI and change in BMI (ΔBMI = BMI T2−BMI T0) were calculated. BMI z-Scores were calculated according to Cole et al. [27]. Parental weight (kilograms) and height (meters) were self-reported at both T0 and T2, and BMI and change in BMI (ΔBMI = BMI T2 − BMI T0) were calculated.

### 2.7. Parental Education

The educational level of the parent included in the present study was considered. It was obtained by questionnaire, and responses could range from “less than six years” to “more than 16 years” of education, with a six-point scale response option.

### 2.8. Statistical Analysis

Normal distribution of variables was tested with Shapiro–Wilk tests and, given that all continuous variables were not normally distributed, Mann–Whitney U tests were performed to compare mean and standard deviations according to children’s and parents’ gender, respectively. Categorical data were analyzed with Chi-square according to parents’ and children’s gender.

For dietary intake variables, range categories in times per week (t/w) and times per day (t/d) of the nine food intake items were recorded to reflect daily intake of servings (s/d) prior to data analyses (less than 1 t/w = 0.14 s/d, 1–2 t/w = 0.21 s/d, 3–4 t/w = 0.5 s/d, 5–6 t/w = 0.79 s/d, 1–2 t/d = 1.5 s/d, 3–4 t/d = 3.5 s/d, 5–6 t/d = 5.5 s/d, and >6 t/d = 6 s/d). For regression analyses, dietary intake variables (s/d) were log-transformed to reduce the effect of their skewness. Individual linear regressions tested baseline associations between FPPs and dietary intake cross-sectionally and the associations between the changes in FPPs from T0 to T2 (ΔFPP) with the changes in dietary intake (Δdietary intake) over time. Regressions were performed adjusting for country, group (intervention-control), parental education, age, gender, and BMI and children’s age, gender, and BMI. Since sex interactions were observed in the associations between FPPs and dietary intake, analyses were stratified by sex.

In order to quantify the probability of complying with current recommendations according to changes or persistence in the use of FPPs, multilevel ordinal logistic regression analyses introducing group (control vs. intervention) and country as levels to account for the study design and adjusted for parental education and children’s and parents’ age, gender, and ΔBMI were performed. Three categories of longitudinal changes in FPPs were introduced as the independent variables. For positive FPP variables, the categories were never/decreased (reference), improved (increased), and often/sometimes at both time points. For negative FPP variables, the categories were often/increased (reference), improved (decreased), and never at both time points. Compliance with recommendations for each food item (yes/no) were considered as dependent variables.

Statistical analyses were performed using the Statistical Package for the Social Sciences (IBM SPSS Statistics for Windows, Version 26.0. Armonk, NY: IBM Corp, USA), except for the multilevel logistic regression model, which was conducted using Stata/SE 13 (Stata Corp LP, College Station, TX, USA). The results were considered significant at *p* < 0.05.

## 3. Results

Demographic and anthropometric characteristics of children and their parents and children’s dietary intake at T0 are presented in Table 1. Regarding compliance with recommendations presented in Supplementary Table S2, results indicated that the majority of children (92.7%) did not comply with recommendations for total fruit and vegetable and water intake (84.0%). Similarly, a large number of children exceeded the recommended amount of sweets (90.9%) and salty snacks and fast food (62.3%).

### 3.1. Cross-Sectional Associations between FPP and Dietary Intake at Baseline

Positive associations between several positive FPPs, such as parental role modeling of fruit intake and fruit and vegetable intake, were observed, whereas negative associations were observed with energy-dense foods, such as soft drinks and salty snacks (Supplementary Tables S3 and S4). The strongest associations were observed between parental role modeling of fruit intake and children’s dietary intake of fruit and home availability of salty snacks and fast food and corresponding intake.

### 3.2. Longitudinal Associations between Changes in FPPs and Dietary Intake over Time

Results from individual linear regressions indicated that changes in positive FPPs (e.g., home availability of fruit or parental role modeling of fruit intake) were positively associated with higher consumption of fruits and berries and with the consumption of fruits and vegetables but not with changes in energy-dense/nutrient-poor food intake (Table 2 and Table 3). The strongest associations were observed between an increase in home availability of 100% fruit juice and soft drinks with corresponding positive change in the intake of such beverages, in addition to the association between change in parental modeling of fruit intake over time and fruit and vegetable intake for both boys and girls. Few differences were observed between boys and girls. The differences that were observed were that home availability of soft drinks was negatively associated with water intake (β = −0.054, *p* < 0.001) and home availability of 100% fruit juice was negatively associated with soft drinks and sugary juices (β = −0.111, *p* < 0.001) in boys but not in girls.

### 3.3. Associations between Changes in FPPs and Compliance with Dietary Recommendations for Water and Nutrient-Dense Foods

Results from multilevel logistic regressions showed that a decrease in soft drinks availability at home increased the odds of girls complying with water consumption recommendations (Table 4 and Table 5). The frequent use of positive FPP was associated with a higher probability of compliance with fruit intake recommendations in all children, and an increase in their use over time was associated with higher odds of complying with recommendations for fruit intake in boys. Regarding vegetable consumption, the frequent presence of fruit and vegetables at home and parental modeling of fruit intake appeared to be significantly associated with compliance with recommendations. Nevertheless, improvements in these practices over time were not associated with the consumption of the recommended amounts of these foods. On the contrary, the persistent avoidance of negative FPPs such as home availability of sugary juices and soft drinks and being permissive in girls was associated with higher odds of complying with consuming one to two servings per day of fruits and vegetables. Also, in girls, changes in negative FPPs over time were not associated with the compliance with water or nutrient-dense food intake recommendations. Conversely, in boys, a decrease in home availability of sweets was associated with 52% higher odds of complying with water intake recommendations. Moreover, never having used food as a reward or a decrease in this practice increased the odds of boys complying with fruit and vegetable recommendations.

### 3.4. Associations between Changes in FPP and Compliance with Dietary Recommendations for Energy-Dense/Nutrient-Poor Foods

Results from multilevel logistic regressions indicated that the use of positive FPPs (Table 6 and Table 7) was not associated with water or nutrient-dense food intake, except for parental modeling of fruit intake, which was associated with 33% higher odds of boys consuming salty snacks and fast food once or less per week. On the other hand, regarding changes in negative FPP, a decrease in the home availability of sugar-sweetened beverages was associated with the consumption of sugary juices and soft drinks once or less per week. Home availability of sweets and salty snacks and fast food was also associated with children’s corresponding intake of such foods. In short, most of the negative FPPs avoided over time were associated with higher odds of complying with the recommendation of limiting the consumption of energy-dense/nutrient-poor foods.

## 4. Discussion

The present study shows that FPPs are associated both cross-sectionally and longitudinally with dietary intake and food intake recommendations compliance of European children from the Feel4Diabetes study. Positive FPPs seem better for the pursuit of compliance with nutrient-dense food recommendations, whereas avoiding negative FPPs appears to be beneficial in limiting the consumption of energy-dense/nutrient-poor foods. It is worth mentioning that these associations were found independently of group (control vs. intervention), parental educational level, gender, sex, and age and children’s gender, sex, and age.

### 4.1. Home Food Availability

Previous research found cross-sectional associations between home food availability and children’s dietary intake of nutrient-dense foods [28,29] and soft drinks [30,31,32,33], which is in line with the associations found in the present study. On the other hand, home availability of energy-dense/nutrient-poor foods was associated with dietary intake of corresponding foods, especially with soft drinks, sweets, and salty snacks, and negatively associated with nutrient-dense food intake. It is worth mentioning that home food availability does not always determine the consumption of corresponding foods, given the findings of previous research that high-calorie/nutrient-poor food availability was negatively associated with its consumption, whereas no association was found between low-calorie/nutrient-dense food availability and F and V consumption [33]. Indeed, we found that a decrease in home availability of sugary juices was negatively associated with water intake, indicating that the presence of some foods may replace the intake of others. Some parental factors may determine home food availability, such as maternal concern for healthy eating [34], preferences [35], and family income [36], so the assessment of such variables would also be relevant when trying to improve the home availability of specific foods.

The predictive value of home availability of food, also known as covert control, on fruit intake has been evaluated by a few studies. For instance, Sleddens et al. [37] observed a positive longitudinal association between parental covert control and the fruit and water intake of their six- to eight-year-old children. According to our results, to comply with recommendations for children’s intake of fruits and vegetables, the regular use of positive practices, such as parental role modeling of fruit intake, is essential. Nevertheless, a decrease in the availability of nutrient-dense foods was significatively associated with recommendation compliance in boys.

Furthermore, an increase in 100% fruit juice was associated with a reduction in soft drink and sugar juice intake, which indicates that a positive replacement of beverages at home may have occurred. On the contrary, a decrease in sugar juice and soft drink availability over time was associated with higher odds of consuming the recommended amount of water, which indicates that the replacement of beverages at home is not only associated with an increase in water consumption but also with a higher probability of complying with recommendations.

Increases in negative FPPs were mainly associated with an increase in the consumption of sweets and salty snacks and fast food, indicating that their presence at home, their use as a reward, and being permissive about their consumption is not positive for children in the long term. Contrary to the associations found between positive FPP and energy-dense/nutrient-poor foods, no associations were observed between improvements in negative FPPs and nutrient-dense food intake (e.g., improvement in modeling fruit and sweets intake). Nevertheless, several positive associations were observed between improvements in the use of FPPs and higher odds of eating energy-dense/nutrient-poor foods once or less per week. These findings indicate the importance of ensuring the availability of nutrient-dense foods at home, besides avoiding the presence of energy-dense/nutrient-poor foods.

### 4.2. Parental Modeling of Fruit Intake

Previously, several studies have confirmed cross-sectional associations between parental modeling of fruit and vegetable intake and children’s dietary intake [33,38,39,40,41,42]. Indeed, we found positive associations between the use of this practice and the consumption of nutrient-dense foods, whereas negative associations were found with the intake of sugar-sweetened beverages, sweets, and salty snacks and fast food.

Not surprisingly, an increase in parental modeling of fruit intake over time was associated with a higher probability of children’s compliance with recommendations for water and fruit and berry intake over time. It is worth considering that modeling food behaviors includes parental modeling of both healthy and unhealthy food choices [43]. Even though in this study we only evaluated modeling of fruit intake, it should be considered that parental modeling of energy-dense/nutrient-poor foods, such as soft drinks and snacks, may also be associated with corresponding dietary intake, as observed in children [44].

Breakfast, snack, and dinner times are good examples of occasions that parents may try to be role models for nutrient-dense food intake for their children by eating fruit, for example. For this reason, it could be useful to help parents identify when they share eating occasions with their children, as those moments may be ideal for role modeling of nutrient-dense foods. Although children may be unaware of their parents’ efforts to role model positive dietary behaviors, such as consuming fruit in front of them, they seem to benefit from a home food environment that provides healthy food options as well as caregivers that make efforts to shape healthy behaviors in their children. Results from previous studies [28,45] and this study showed that it is one of the FPPs that showed the strongest associations with children’s dietary intake, especially fruit intake.

### 4.3. Permissiveness: Allowance of Sweets and Salty Snacks

Previous studies have found significant associations between permissiveness and dietary intake of energy-dense/nutrient-poor foods such as unhealthy snacks and soft drinks in European preschoolers [12] and school-age children [32,46]. Our study confirms previous findings, given that permissiveness appeared to be associated with higher consumption of the four energy-dense/nutrient-poor foods assessed and lower intake of water, fruits, and vegetables.

Even though several associations were observed between the use of this practice and dietary intake at baseline, an increase in its use over time was only associated with lower fruit consumption and higher sweets and salty snack and fast-food consumption. Regarding dietary intake compliance in those whose parents decreased the use of this practice over time, no associations were observed, except for a negative association with the consumption of 100% fruit juice.

Moderate restriction has been identified as a positive FPP since it helps parents to give clearer instructions to their children [47]; in this sense, the assessment of both practices and their interaction would be important to determine the degree of influence that each one has on children’s intake of energy-dense/nutrient-poor foods.

### 4.4. Use of Food as A Reward

The use of food-based rewards or incentives can be used by parents either to manage children’s eating behaviors or to improve their behavior [10]. This may have a negative long-term impact on children in terms of emotional feeding [48] and picky eating [49] as it is associated with a decreased liking of target foods [50]. On the other hand, items used as rewards are commonly unhealthy [51] and it is, therefore, an important determinant of their consumption. As with the previously mentioned FPPs, several associations were observed between the use of this practice and dietary intake at baseline, but an increase in its use over time was only associated with higher sweets and salty snack and fast-food consumption.

However, no studies evaluating associations between the use of food as a reward and dietary intake have been published. As expected from baseline associations, our findings indicated that a decrease in the use of this practice had no associations with nutrient-dense food intake, but it was shown to be significantly associated with less salty snacks and fast food consumption per week, which indicates that modifications in the use of this practice directly affect salty snack and fast-food intake. Thus, as concluded by a previous study [49], the best advice for parents would be to limit the use of food as a reward and to encourage them to motivate children with non-nutritional rewards.

It is worth mentioning that the majority of children presented a high consumption of sugary juices and soft drinks, which is relevant because these beverages and foods have been identified as the main sources of free sugars and energy at young ages [52,53,54]. Their consumption has been associated with the development of excessive weight, dislypemia, and insulin resistance [55]. Fortunately, improvements in FPPs, such as reducing the availability of soft drinks and sweets and avoiding the use of food as a reward, increase the likelihood that children will consume such foods once or less per week, thus meeting current recommendations.

It should be noted that, although FPPs are individually significantly associated with the intake of certain foods, parents employ multiple practices with their children, which means that co-occurrence of positive and negative FPPs may exist [56]. Therefore, it could be beneficial to establish FPP patterns or clusters that could better identify the types of FPPs employed by parents. Also, future studies should consider assessing FPPs in both parents and main caregivers to evaluate if concordance or discordance between these practices in all the members of the family may affect the observed associations with children’s dietary intake. Social characteristics of children may also be of relevance, given that, for example, children from divorced parents may be exposed to more than one home food environment and FPP.

### 4.5. Strengths and Limitations

Important strengths of the Feel4Diabetes study include the longitudinal nature of the design and the fact that standardized protocols and procedures were followed across all centers. Observed changes cannot be attributable to the Feel4Diabetes study intervention group because control data was also available and the variable group (control vs. intervention) was included in the models to account for random effects. Also, among the strengths of the present study is the fact that our sample includes a large and socioeconomically diverse population of primary-school-aged children and their families from six European countries. However, some limitations of this study must be considered. Although the questionnaire reliability was tested in volunteers in each country before the study, [24] a food frequency questionnaire was used to assess usual diet, and as with any assessment of dietary intake, underreporting of usual intake or invalid reporting due to social desirability bias is possible, especially because it was self-reported by parents, which may have introduced self-report bias [57].

As mentioned previously, children’s eating behavior is the result of multiple levels of influence, so, even though we have tried to focus on modifiable factors in this study, the results must be interpreted with caution. Also, 40% of parents included in this study were low educated. This limits the generalizability of our results because of misreporting bias, which appears to be more common in populations of lower than higher socioeconomic status [58]. The results of this study should also be taken with caution due to the fact that reporting of this practice differ slightly depending on who reports the information (e.g., the parent or the child), and it may therefore be different if children are the ones who report their perceptions regarding their parents’ role as models of fruit intake. Also, we must consider that the reporting parent is not the only person involved in shaping their child’s dietary habits and that the information about other members of the family, e.g., the other parent, may also affect children’s dietary intake [42].

## 5. Conclusions

In general, our results indicate that the more positive FPPs and the fewer negative FPPs used, the higher the odds of children complying with recommendations for nutrient-dense and energy-dense/nutrient-poor foods. Dietitians and health carers should assess FPPs in order to target environmental surroundings, to make improvements in the quality of foods available in families’ homes and to encourage parents to be role models of nutrient-dense food intake for their children and avoid negative FPP such as permissiveness or the use of food as a reward. These findings shed light on the potential FPPs that future interventions should focus on to improve children’s dietary intake. Nevertheless, more longitudinal studies are needed to assess the prospective impact of FPPs over time.

## Figures and Tables

**Table 1:** Characteristics at baseline (T0) from the study participants; *n* = 2967 *.

Children	All	Girls	Boys	*p*
Demographics, % (*n*) Age (y), mean ± SD Weight (kg), mean ± SD Height (cm), mean ± SD BMI, mean ± SD z-BMI, mean ± SD	2967 * 8.09 ± 0.93 29.13 ± 6.59 130.28 ± 7.72 17.01 ± 2.60 0.48 ± 1.04	50.4 (1494) 8.10 ± 0.93 28.88 ± 6.54 129.84 ± 7.66 16.98 ± 2.63 0.49 ± 1.02	49.6 (1473) 8.09 ± 0.94 29.37 ± 6.63 130.70 ± 7.75 17.03 ± 2.58 0.48 ± 1.05	- 0.919 **0.030** **0.002** 0.493 0.781
Country, % (*n*) Belgium Bulgaria Finland Greece Hungary Spain	20.4 (606) 22.5 (669) 15.6 (462) 21.5 (637) 6.4 (189) 13.6 (404)	19.1 (285) 23.8 (356) 15.7 (235) 21.5 (321) 7.2 (107) 12.7 (190)	21.8 (321) 21.2 (313) 15.4 (227) 21.5 (316) 5.6 (82) 14.5 (214)	0.085
Dietary intake servings/day, Water Fresh fruits and berries Canned fruits and berries 100% fruit juice Vegetables Fruits and vegetables ^1^ Fruits and vegetables ^2^ Soft drinks and sugar juices Light soft drinks Sweets Salty snacks and fast food ^†^	3.84 ± 1.71 1.18 ± 0.75 0.21 ± 0.23 0.48 ± 0.54 1.03 ± 0.69 2.68 ± 1.27 2.89 ± 1.31 0.35 ± 0.53 0.19 ± 0.33 0.79 ± 0.64 0.29 ± 0.36	3.78 ± 1.71 1.21 ± 0.79 0.22 ± 0.23 0.45 ± 0.51 1.05 ± 0.70 2.71 ± 1.27 2.93 ± 1.31 0.31 ± 0.45 0.19 ± 0.27 0.76 ± 0.63 0.28 ± 0.32	3.91 ± 1.70 1.14 ± 0.71 0.21 ± 0.22 0.51 ± 0.57 1.00 ± 0.68 2.65 ± 1.27 2.86 ± 1.30 0.38 ± 0.61 0.20 ± 0.39 0.81 ± 0.66 0.31 ± 0.39	**0.029** 0.077 0.134 **0.036** 0.063 0.310 0.270 **<0.001** 0.168 **0.032** **0.008**
Parents	**All**	**Mothers**	**Fathers**	***p***
Demographics, % (*n*) Parental education, % (*n*) <6 y 7–9 y 10–12 y 13–14 y 15–16 y >16 y Age (y), mean ± SD BMI (kg/m^2^), mean ± SD	2967 0.3 (9) 1.8 (52) 17.1 (507) 13.7 (406) 31.1 (924) 36.0 (1069) 38.61 ± 4.67 23.94 ± 4.29	93.5 (2773) 0.3 (9) 1.7 (47) 16.7 (464) 13.5 (373) 31.8 (881) 36.0 (999) 38.39 ± 4.53 23.77 ± 4.29	6.5 (194) 0 2.6 (5) 22.2 (43) 17.0 (33) 22.2 (43) 36.1 (70) 41.85 ± 5.36 26.36 ± 3.58	- **0.040** **<0.001** **<0.001**
Group, % (*n*) Control Intervention Country, % (*n*) Belgium Bulgaria Finland Greece Hungary Spain	49.1 (1456) 50.9 (1511) 20.4 (606) 22.5 (669) 15.6 (462) 21.5 (637) 6.4 (189) 13.6 (404)	49.0 (1358) 51.0 (1415) 19.7 (546) 23.5 (651) 15.3 (424) 21.1 (584) 6.7 (186) 13.8 (382)	50.5 (98) 49.5 (96) 30.9 (60) 9.3 (18) 19.6 (38) 27.3 (53) 1.5 (3) 11.3 (22)	0.678 **<0.001**

*n* = 2967 *, except for salty snacks ^†^, *n* = 2500. Boldface indicates statistical significance between sexes at *p* < 0.05. Chi-square test was used to test differences by sex for categorical data. Mann–Whitney U tests were performed to test differences by sex in continuous variables. Abbreviations: z-BMI, body mass index z-score according to Cole et al. (2010); BMI, body mass index; y, years; SD, standard deviation. Fruits and vegetables^1^: fresh or frozen fruit and berries, fresh fruit juices, and vegetables. Fruits and vegetables^2^: Same as in Fruits and vegetables^1^ and together with canned fruits and berries.

**Table 2:** Associations between changes from baseline to year two (Δ = T2 − T0) of food parenting practices and changes in dietary intake in girls.

	Nutrient-Dense Foods							Energy-Dense/Nutrient-Poor Foods			
	Δ Water	Δ Fruits and Berries	Δ Canned Fruits and Berries	Δ 100% Fruit Juice	Δ Vegetables	Δ F and V^1^	Δ F and V^2^	Δ Soft Drinks and Sugar Juices	Δ Light Soft Drinks	Δ Sweets	Δ Salty Snacks and Fast Food
	β (*p*-Value)	β (*p*-Value)	β (*p*-Value)	β (*p*-Value)	β (*p*-Value)	β (*p*-Value)	β (*p*-Value)	β (*p*-Value)	β (*p*-Value)	β (*p*-Value)	β (*p*-Value)
Positive FPP											
Δ HA Fruit	- 0.030 (0.061)	**0.132 (<0.001)**	**−0.051 (0.046)**	0.011 (0.655)	**0.058 (0.024)**	**0.129 (<0.001)**	**0.115 (<0.001)**	−0.032 (0.193)	0.012 (0.598)	−0.006 (0.819)	−0.006 (0.821)
Δ HA 100% fruit juice	0.008 (0.625)	**0.049 (0.030)**	0.042 (0.100)	**0.228 (<0.001)**	**0.014 (0.600)**	**0.102 (<0.001)**	**0.106 (<0.001)**	0.032 (0.191)	−0.015 (0.497)	0.015 (0.533)	−0.015 (0.586)
Δ HA Vegetables	0.011 (0.493)	**0.074 (0.001)**	−0.022 (0.397)	0.033 (0.176)	**0.111 (<0.001)**	**0.121 (<0.001)**	**0.116 (<0.001)**	−0.005 (0.842)	−0.001 (0.963)	0.020 (0.415)	−0.009 (0.759)
Δ Modeling of fruit intake	**0.038 (0.020)**	**0.165 (<0.001)**	0.049 (0.059)	0.036 (0.141)	**0.093 (<0.001)**	**0.174 (<0.001)**	**0.180 (<0.001)**	**−0.069 (0.005)**	−0.006 (0.776)	0.018 (0.466)	−0.028 (0.328)
Negative FPP											
Δ HA Sugar juices	0.001 (0.935)	**−0.054 (0.019)**	0.024 (0.349)	0.033 (0.180)	−0.019 (0.467)	−0.018 (0.486)	−0.011 (0.664)	**0.197 (<0.001)**	**−0.045 (0.043)**	**0.082 (0.001)**	0.049 (0.080)
Δ HA Soft drinks	0.000 (0.986)	−0.013 (0.564)	0.039 (0.126)	0.013 (0.597)	**−0.070 (0.007)**	−0.030 (0.242)	−0.020 (0.453)	**0.147 (<0.001)**	**−0.055 (0.014)**	0.029 (0.239)	**0.118 (<0.001)**
Δ HA Light soft drinks	0.020 (0.230)	−0.031 (0.171)	0.029 (0.266)	−0.001 (0.977)	0.009 (0.724)	−0.011 (0.665)	−0.007 (0.782)	−0.004 (0.886)	**0.066 (0.003)**	0.009 (0.709)	**0.058 (0.040)**
Δ HA Sweets	0.012 (0.484)	−0.031 (0.171)	−0.010 (0.705)	−0.022 (0.377)	−0.017 (0.504)	−0.027 (0.312)	−0.027 (0.310)	**0.063 (0.010)**	0.008 (0.727)	**0.186 (<0.001)**	**0.145 (<0.001)**
Δ HA Salty snacks	0.018 (0.266)	0.017 (0.462)	−0.030 (0.425)	0.017 (0.493)	−0.018 (0.475)	0.013 (0.603)	0.012 (0.655)	0.037 (0.128)	−0.028 (0.210)	**0.123 (<0.001)**	**0.221 (<0.001)**
Δ Permissiveness	0.022 (0.177)	−0.039 (0.092)	−0.066 (0.011)	0.046 (0.060)	−0.042 (0.107)	−0.034 (0.189)	−0.041 (0.113)	0.021 (0.384)	−0.018 (0.422)	**0.023 (<0.001)**	**0.077 (0.006)**
Δ Use of foods as reward *	−0.018 (0.270)	0.009 (0.695)	0.007 (0.772)	0.024 (0.320)	**−0.056 (0.031)**	−0.003 (0.907)	−0.003 (0.919)	0.017 (0.484)	0.008 (0.735)	**0.104 (<0.001)**	**0.074 (0.009)**

*n* = 1494, except salty snacks *n* = 1268. Linear regressions were performed individually and were adjusted for country, group (control vs. intervention), parental education, sex, age, and the change from T0 to T2 of BMI and children’s gender, age, and the change from T0 to T2 in BMI. β = Standardized coefficients; FPP, food parenting practices; HA, home availability. Boldface indicates statistical significance at 0.05. (*) Unhealthy foods such as sweets, salty snacks, and fast food.

**Table 3:** Associations between changes from baseline to year two (Δ = T2 − T0) of food parenting practices and changes in dietary intake in boys.

	Nutrient-Dense Foods							Energy-Dense/Nutrient-Poor Foods			
	Δ Water	Δ Fruits and Berries	Δ Canned Fruits and Berries	Δ 100% Fruit Juice	Δ Vegetables	Δ F and V^1^	Δ F and V^2^	Δ Soft Drinks and Sugar Juices	Δ Light Soft Drinks	Δ Sweets	Δ Salty Snacks and Fast Food
	β (*p*-Value)	β (*p*-Value)	β (*p*-Value)	β (*p*-Value)	β (*p*-Value)	β (*p*-Value)	β (*p*-Value)	β (*p*-Value)	β (*p*-Value)	β (*p*-Value)	β (*p*-Value)
Positive FPP											
Δ HA Fruit	−0.001 (0.955)	**0.158 (<0.001)**	−0.011 (0.662)	0.031 (0.208)	0.008 (0.764)	**0.101 (<0.001)**	**0.097 (<0.001)**	−0.020 (0.441)	−0.005 (0.837)	−0.006 (0.810)	−0.015 (0.610)
Δ HA 100% fruit juice	−0.025 (0.152)	**0.032 (0.185)**	0.024 (0.348)	**0.339 (<0.001)**	**0.014 (0.602)**	**0.153 (<0.001)**	**0.156 (<0.001)**	**−0.111 (<0.001)**	0.027 (0.278)	−0.029 (0.251)	0.006 (0.836)
Δ HA Vegetables	−0.007 (0.673)	**0.094 (<0.001)**	0.023 (0.371)	0.004 (0.863)	**0.096 (<0.001**)	**0.119 (<0.001)**	**0.121 (<0.001)**	0.018 (0.475)	−0.007 (0.788)	**0.049 (0.049)**	−0.012 (0.672)
Δ Modeling of fruit intake	0.021 (0.234)	**0.216 (<0.001)**	0.007 (0.778)	0.017 (0.489)	0.050 (0.056)	**0.131 (<0.001)**	**0.128 (<0.001)**	−0.032 (0.205)	0.020 (0.408)	−0.042 (0.094)	0.011 (0.707)
Negative FPP											
Δ HA Sugar juices	−0.029 (0.101)	−0.012 (0.618)	0.039 (0.137)	0.045 (0.071)	−0.019 (0.466)	0.014 (0.603)	0.021 (0.433)	**0.169 (<0.001)**	0.038 (0.128)	**0.080 (0.002)**	**0.076 (0.008)**
Δ HA Soft drinks	**−0.054 (<0.001)**	**−0.057 (0.022)**	0.025 (0.333)	0.041 (0.093)	−0.010 (0.689)	−0.020 (0.444)	−0.012 (0.656)	**0.095 (<0.001)**	0.036 (0.146)	**0.056 (0.026)**	**0.070 (0.015)**
Δ HA Light soft drinks	−0.018 (0.298)	0.002 (0.932)	0.002 (0.940)	**0.056 (0.021)**	0.020 (0.444)	0.047 (0.074)	0.046 (0.080)	0.015 (0.546)	**0.095 (<0.001)**	0.041 (0.102)	**0.064 (0.026)**
Δ HA Sweets	0.005 (0.754)	−0.004 (0.855)	−0.009 (0.724)	0.019 (0.441)	0.024 (0.363)	0.025 (0.332)	0.022 (0.401)	−0.003 (0.892)	−0.020 (0.421)	**0.213 (<0.001)**	**0.103 (<0.001)**
Δ HA Salty snacks	**−0.045 (0.010)**	−0.009 (0.727)	−0.035 (0.184)	−0.002 (0.940)	−0.001 (0.961)	0.013 (0.629)	0.007 (0.799)	0.015 (0.546)	−0.026 (0.290)	**0.067 (0.008)**	**0.190 (<0.001)**
Δ Permissiveness	−0.032 (0.067)	−0.046 (0.058)	−0.026 (0.319)	0.038 (0.118)	−0.013 (0.629)	−0.018 (0.502)	−0.018 (0.485)	0.014 (0.574)	0.005 (0.829)	**0.096 (<0.001)**	**0.078 (0.006)**
Δ Use of foods as reward *	0.002 (0.909)	0.016 (0.503)	0.014 (0.582)	−0.008 (0.759)	−0.036 (0.164)	−0.016 (0.541)	−0.011 (0.663)	0.047 (0.067)	0.031 (0.202)	**0.038 (0.135)**	**0.073 (0.010)**

*n* = 1473, except salty snacks *n* = 1232. Linear regressions were performed individually and were adjusted for country, group (control vs. intervention), parental education, sex, age, and the change from T0 to T2 of BMI and children’s gender, age, and the change from T0 to T2 in BMI. β = Standardized coefficients; FPP, food parenting practices; HA, home availability. Boldface indicates statistical significance at 0.05. (*) Unhealthy foods such as sweets, salty snacks, and fast food.

**Table 4:** Multilevel logistic regression analysis by combinations of change for food parenting practices over time (T0 to T2) and its effects on the compliance of recommendations for healthy foods at follow-up in girls, *n =* 1494.

	Categories of Nutrient-Dense Foods ^†^						
	Water	Fruits and Berries	Canned Fruits	100% Fruit Juice	Vegetables	F and V^1^	F and V^2^
	OR (95% CI)	OR (95% CI)	OR (95% CI)	OR (95% CI)	OR (95% CI)	OR (95% CI)	OR (95% CI)
**Positive Food Parenting Practices**							
HA of fruit Never/Decreased Improved (increased) Often	Ref. 1.90 (0.44; 8.24) 2.66 (0.79; 8.96)	Ref. 3.04 (0.85; 10.90) **11.42 (3.93; 33.15)**	Ref. N.A 1.04 (0.13; 8.07)	Ref. 2.09 (0.39; 11.08) 2.41 (0.64; 9.08)	Ref. 2.13 (0.66; 6.89) **5.25 (2.10; 13.15)**	N.A	N.A
HA of 100% fruit juice Never/Decreased Improved Often	Ref. 1.15 (0.75; 1.77) 1.22 (0.87; 1.71)	Ref. 1.05 (0.76; 1.45) **1.29 (1.00; 1.66)**	Ref. **2.78 (1.09; 1.11)** 1.98 (0.84; 4.66)	Ref. **1.91 (1.05; 3.45)** **3.54 (2.18; 5.75)**	Ref. 0.92 (0.67; 1.26) 1.01 (0.78; 1.30)	Ref. 0.82 (0.46; 1.44) 0.71 (0.45; 1.12)	Ref. 1.05 (0.62; 1.78) 0.87 (0.56; 1.33)
HA of vegetables Never/Decreased Improved Often	Ref. 0.35 (0.09; 1.45) 0.99 (0.46; 2.13)	Ref. 1.46 (0.54; 3.98) **3.31 (1.68; 6.51)**	Ref. 1.28 (0.08; 21.81) 1.12 (0.15; 8.64)	Ref. 1.20 (0.34; 4.27) 1.38 (0.56; 3.42)	Ref. 2.37 (0.76; 7.43) **5.09 (2.21; 11.77)**	N.A	N.A
Modeling of fruit Never/Decreased Improved Often	Ref. 1.35 (0.83; 2.19) 1.38 (0.96; 2.00)	Ref. 1.28 (0.89; 1.84) **2.90 (2.21; 3.80)**	Ref. 1.13 (0.34; 3.69) 1.15 (0.48; 2.76)	Ref. 1.77 (0.97; 3.22) **1.88 (1.17; 3.03)**	Ref. 1.06 (0.73; 1.53) **1.75 (1.33; 2.29)**	Ref. 1.69 (0.76; 3.76) **3.01 (1.64; 5.51)**	Ref. 1.49 (0.69; 3.21) **2.93 (1.66; 5.16)**
**Negative Food Parenting Practices**							
HA of sugar juices Often/Increased Improved (decreased) Never	Ref. 1.07 (0.73; 1.57) 0.99 (0.69; 1.41)	Ref. 0.98 (0.74; 1.31) **1.39 (1.07; 1.82)**	Ref. 1.00 (0.40; 2.50) 1.00 (0.44; 2.22)	Ref. 0.62 (0.38; 1.02) 0.98 (0.64; 1.50)	Ref. 1.07 (0.80; 1.44) **1.52 (1.16; 1.99)**	Ref. 1.18 (0.69; 2.03) 1.53 (0.95; 2.48)	Ref. 1.14 (0.68; 1.89) 1.41 (0.90; 2.23)
HA of soft drinks Often/Increased Improved (decreased) Never	Ref. **1.57 (1.01; 2.45)** 1.10 (0.76; 1.60)	Ref. 1.21 (0.85; 1.71) **1.34 (1.02; 1.77)**	Ref. 1.22 (0.39; 3.85) 1.27 (0.53; 3.09)	Ref. 1.15 (0.67; 1.94) 0.91 (0.59; 1.42)	Ref. 1.11 (0.78; 1.59) **1.58 (1.19; 2.09)**	Ref. 0.74 (0.40; 1.37) 0.80 (0.50; 1.26)	Ref. 0.85 (0.47; 1.52) 0.84 (0.54; 1.30)
HA of light soft drinks Often/Increased Improved (decreased) Never	Ref. 1.54 (0.96; 2.47) 0.99 (0.69; 1.42)	Ref. 1.06 (0.72; 1.57) 0.95 (0.72; 1.24)	Ref. 1.47 (0.41; 5.22) 1.29 (0.49; 3.38)	Ref. 0.63 (0.34; 1.16) 0.74 (0.49; 1.11)	Ref. 1.03 (0.69; 1.54) 1.05 (0.80; 1.38)	Ref. 1.88 (0.99; 3.55) 1.41 (0.86; 2.31)	Ref. **1.92 (1.04; 3.54)** 1.38 (0.86; 2.21)
HA of sweets Often/Increased Improved (decreased) Never	Ref. 0.99 (0.69; 1.42) 1.37 (0.48; 3.91)	Ref. 0.93 (0.71; 1.23) 1.36 (0.61; 3.04)	Ref. 2.04 (0.97; 4.30) 1.48 (0.18; 12.00)	Ref. 1.04 (0.66; 1.64) 1.58 (0.43; 5.73)	Ref. 1.11 (0.84; 1.48) 0.99 (0.44; 2.25)	Ref. 1.07 (0.64; 1.79) 0.63 (0.08; 4.83)	Ref. 1.12 (0.70; 1.81) 0.49 (0.06; 3.73)
HA of salty snacks Often/Increased Improved (decreased) Never	Ref. 1.35 (0.93; 1.97) 0.89 (0.60; 1.33)	Ref. 0.94 (0.70; 1.27) 1.21 (0.91; 1.62)	Ref. 2.44 (0.95; 6.29) **3.45 (1.50; 7.90)**	Ref. 0.91 (0.56; 1.47) 0.68 (0.41; 1.14)	Ref. 1.09 (0.81; 1.48) 1.11 (0.83; 1.48)	Ref. 0.87 (0.49; 1.55) 1.20 (0.71; 2.00)	Ref. 1.12 (0.66; 1.88) 1.41 (0.87; 2.27)
Permissiveness Often/Increased Improved (decreased) Never	Ref. 1.14 (0.79; 1.64) 0.83 (0.56; 1.22)	Ref. 1.01 (0.76; 1.35) **1.37 (1.04; 1.81)**	Ref. 1.23 (0.50; 3.02) 1.41 (0.62; 3.22)	Ref. 0.96 (0.60; 1.53) 0.80 (0.49; 1.30)	Ref. 1.18 (0.88; 1.58) **1.44 (1.09; 1.91)**	Ref. 1.08 (0.65; 1.81) 0.98 (0.59; 1.63)	Ref. 1.13 (0.70; 1.84) 1.08 (0.67; 1.74)
Use of food as a reward * Often/Increased Improved (decreased) Never	Ref. 0.93 (0.53; 1.63) 1.10 (0.72; 1.65)	Ref. 0.74 (0.49; 1.11) 1.11 (0.83; 1.50)	Ref. 1.01 (0.29; 3.51) 0.79 (0.31; 2.03)	Ref. 0.53 (0.26; 1.11) 0.82 (0.52; 1.30)	Ref. 0.79 (0.52; 1.21) 1.21 (0.89; 1.63)	Ref. 0.64 (0.31; 1.36) 0.76 (0.46; 1.24)	Ref. 0.69 (0.34; 1.40) 0.76 (0.47; 1.21)

*n* = 1494. Abbreviations: OR, odds ratio (odds for being allocated in the group that follows recommendations); 95% CI, 95% confidence interval. T0, baseline period, T2, follow-up period, CI, confidence intervals; Ref, reference category; HA, home availability; N.A, not applicable. F and V^1^: indicates the sum of servings of fresh or frozen fruit and berries, fresh fruit juices, and vegetables. F and V^2^: Same as in fruits and vegetables^1^ and together with canned fruits and berries. Multilevel logistic regression adjusted for parental (age, gender, education level, and change in BMI from T0 to T2) and children’s (age, gender, and change in BMI from T0 to T2) characteristics. Categories of FPP indicate a change in the use of them over time. All models of the multilevel logistic regression include random effects (country and group) to account for the study design. **^†^** Analyses were performed with outcome variables indicating compliance with recommendations, where 0 = no and 1 = yes. Ref. categories for healthy foods were the following: water = six or more servings per day; fruits and berries, canned fruit, fresh fruit juice and vegetables = one to two servings per day, F and V^1^ and F and V^2^ = five or more servings per day. (*) Unhealthy foods such sweets, salty snacks, and fast food.

**Table 5:** Multilevel logistic regression analysis by combinations of change for food parenting practices over time (T0 to T2) and its effects on the compliance of recommendations for healthy foods at follow-up in boys, *n =* 1473.

	Categories of Nutrient-Dense Foods ^†^						
	Water	Fruits and Berries	Canned Fruits	100% Fruit Juice	Vegetables	F and V^1^	F and V^2^
	OR (95% CI)	OR (95% CI)	OR (95% CI)	OR (95% CI)	OR (95% CI)	OR (95% CI)	OR (95% CI)
**Positive Food Parenting Practices**							
HA of fruit Never/Decreased Improved (increased) Often	Ref. 0.38 (0.12; 1.24) 0.73 (0.35; 1.51)	Ref. **4.03 (1.24; 13.12)** **10.02 (3.85; 26.08)**	Ref. N.A 0.95 (0.12; 7.41)	Ref. 0.43 (0.11; 1.75) 1.07 (0.45; 2.52)	Ref. 1.05 (0.35; 3.11) **3.10 (1.47; 6.55)**	Ref. 0.70 (0.06; 8.41) 2.56 (0.54; 12.04)	Ref. 0.72 (0.06; 8.60) 3.12 (0.66; 14.60)
HA of 100% fruit juice Never/Decreased Improved Often	Ref. 1.11 (0.73; 1.70) 0.91 (0.65; 1.28)	Ref. **1.41 (1.02; 1.94)** **1.51 (1.17; 1.95)**	Ref. 1.90 (0.62; 5.83) 2.53 (0.99; 6.44)	Ref. **4.50 (2.57; 7.87)** **8.17 (5.03; 13.28)**	Ref. 1.26 (0.91; 1.76) 1.03 (0.79; 1.35)	Ref. 1.09 (0.63; 1.91) 1.05 (0.68; 1.62)	Ref. 1.53 (0.93; 2.54) 1.30 (0.85; 1.98)
HA of vegetables Never/Decreased Improved Often	Ref. **0.28 (0.08; 0.94)** **0.47 (0.22; 1.00)**	Ref. 1.70 (0.56; 5.18) **3.65 (1.61; 8.25)**	Ref. N.A 0.58 (0.07; 4.58)	Ref. 0.91 (0.23; 3.56) 1.41 (0.53; 3.72)	Ref. 2.51 (0.52; 12.08) **9.51 (2.81; 32.25)**	Ref. 1.99 (0.31; 12.89) 1.74 (0.40; 7.45)	Ref. 1.22 (0.23; 6.59) 1.25 (00.37; 4.19)
Modeling of fruit Never/Decreased Improved Often	Ref. 1.08 (0.66; 1.76) 1.25 (0.88; 1.77)	Ref. **2.20 (1.52; 3.21)** **3.50 (2.66; 4.59)**	Ref. 3.45 (0.80; 14.87) 3.01 (0.87; 10.35)	Ref. 1.22 (0.75; 2.00) 1.05 (0.73; 1.52)	Ref. 1.31 (0.88; 1.94) **1.81 (1.37; 2.39)**	Ref. 1.03 (0.46; 2.30) **2.17 (1.28; 3.70)**	Ref. 1.24 (0.60; 2.56) **2.33 (1.41; 3.85)**
**Negative Food Parenting Practices**							
HA of sugar juices Often/Increased Improved (decreased) Never	Ref. 1.64 (1.14; 2.35) 1.37 (0.96; 1.95)	Ref. 1.08 (0.82; 1.43) 1.23 (0.95; 1.60)	Ref. 0.56 (0.18; 1.80) 1.45 (0.62; 3.37)	Ref. 0.79 (0.54; 1.17) 0.76 (0.53; 1.10)	Ref. 1.24 (0.92; 1.67) 1.30 (0.98; 1.71)	Ref. 0.61 (0.36; 1.01) 0.76 (0.48; 1.19)	Ref. 0.68 (0.42; 1.09) 0.86 (0.56; 1.31)
HA of soft drinks Often/Increased Improved (decreased) Never	Ref. 1.04 (0.67; 1.62) 1.09 (0.76; 1.57)	Ref. 1.27 (0.91; 1.77) **1.57 (1.20; 2.06)**	Ref. 0.53 (0.11; 2.65) 1.79 (0.70; 4.60)	Ref. 0.97 (0.61; 1.54) 1.07 (0.73; 1.55)	Ref. 1.03 (0.72; 1.48) **1.54 (1.16; 2.05)**	Ref. **0.48 (0.24; 0.95)** 1.07 (0.70; 1.64)	Ref. **0.48 (0.25; 0.93)** 1.22 (0.82; 1.83)
HA of light soft drinks Often/Increased Improved (decreased) Never	Ref. 0.74 (0.44; 1.25) 1.13 (0.80; 1.61)	Ref. 0.85 (0.58; 1.25) 1.10 (0.84; 1.44)	Ref. 1.62 (0.39; 6.70) 1.76 (0.64; 4.88)	Ref. 0.78 (0.46; 1.32) 0.84 (0.58; 1.20)	Ref. 0.78 (0.52; 1.19) 1.05 (0.79; 1.39)	Ref. 0.82 (0.39; 1.71) 1.21 (0.78; 1.86)	Ref. 0.92 (0.47; 1.81) 1.31 (0.87; 1.96)
HA of sweets Often/Increased Improved (decreased) Never	Ref. **1.52 (1.07; 2.17)** 0.97 (0.32; 2.99)	Ref. 0.99 (0.73; 1.33) 1.80 (0.83; 3.95)	Ref. 0.56 (0.16; 1.88) N.A	Ref. 0.81 (0.53; 1.25) 0.87 (0.28; 2.68)	Ref. 0.92 (0.67; 1.26) 1.38 (0.63; 3.02)	Ref. 0.78 (0.44; 1.38) 0.88 (0.20; 3.81)	Ref. 0.97 (0.59; 1.60) 0.69 (0.16; 3.01)
HA of salty snacks Often/Increased Improved (decreased) Never	Ref. 1.34 (0.93; 1.92) 1.19 (0.82; 1.74)	Ref. 0.95 (0.71; 1.28) **1.47 (1.09; 1.97)**	Ref. 1.14 (0.43; 3.00) 0.99 (0.35; 2.81)	Ref. 1.33 (0.88; 2.00) **1.67 (1.11; 2.50)**	Ref. 0.96 (0.70; 1.31) 1.27 (0.94; 1.73)	Ref. 1.02 (0.59; 1.77) **1.89 (1.20; 2.99)**	Ref. 1.34 (0.83; 2.16) **1.85 (1.19; 2.88)**
Permissiveness Often/Increased Improved (decreased) Never	Ref. 1.12 (0.78; 1.60) 0.92 (0.62; 1.37)	Ref. 1.01 (0.75; 1.35) 1.12 (0.84; 1.50)	Ref. 0.49 (0.14; 1.68) 0.78 (0.28; 2.16)	Ref. **0.52 (0.34; 0.81)** **0.53 (0.34; 0.83)**	Ref. 0.85 (0.63; 1.16) 1.05 (0.77; 1.42)	Ref. 1.26 (0.76; 2.08) 1.35 (0.83; 2.19)	Ref. 1.17 (0.73; 1.89) 1.33 (0.85; 2.09)
Use of food as a reward * Often/Increased Improved (decreased) Never	Ref. 0.68 (0.41; 1.12) **0.68 (0.46; 0.99)**	Ref. 0.80 (0.55; 1.17) 1.12 (0.83; 1.50)	Ref. 1.08 (0.30; 3.82) 1.00 (0.36; 2.77)	Ref. 1.03 (0.57; 1.87) **1.65 (1.06; 2.57)**	Ref. 1.29 (0.86; 1.94) **1.54 (1.12; 2.11)**	Ref. **2.30 (1.04; 5.08)** **2.43 (1.23; 4.78)**	Ref. 1.53 (0.78; 3.03) 1.71 (0.98; 2.98)

N= 1473. Abbreviations: OR, odds ratio (odds for being allocated in the group that follows recommendations); 95% CI, 95% confidence interval. T0, baseline period, T2, follow-up period, CI, confidence intervals; Ref, reference category; HA, home availability; N.A, not applicable. F and V^1^: indicates the sum of servings of fresh or frozen fruit and berries, fresh fruit juices and vegetables. F and V^2^: Same as in fruits and vegetables^1^ and together with canned fruits and berries. Multilevel logistic regression adjusted for parental (age, gender, education level, and change in BMI from T0 to T2) and children’s (age, gender, and change in BMI from T0 to T2) characteristics. Categories of FPP indicate change in the use of them over time. All models of the multilevel logistic regression include random effects (country and group) to account for the study design. **^†^** Analyses were performed with outcome variables indicating compliance with recommendations, where 0 = no and 1 = yes. Ref. categories for healthy foods were the following: water = six or more servings per day; fruits and berries, canned fruit, fresh fruit juice and vegetables = one to two servings per day, F and V^1^ and F and V^2^ = five or more servings per day. (*) Unhealthy foods such as sweets, salty snacks, and fast food.

**Table 6:** Multilevel logistic regression analysis by combinations of change for food parenting practices over time (T0 to T2) and its effects on the compliance of recommendations for energy-dense/nutrient-poor foods at follow-up in girls, *N* = 1494 *.

	Categories of Energy-Dense/Nutrient-Poor Foods ^†^			
	Soft Drinks and Sugar Juices	Light Soft Drinks	Sweets	Salty Snacks and Fast Foods
	OR (95% CI)	OR (95% CI)	OR (95% CI)	OR (95% CI)
**Positive Food Parenting Practices**				
HA of fruit Never/Decreased Improved (increased) Often	Ref. 1.20 (0.44; 3.26) 0.93 (0.44; 1.93)	Ref. 0.38 (0.11; 1.36) 0.76 (0.28; 2.06)	Ref. 1.22 (0.30; 4.97) 0.82 (0.29; 2.34)	Ref. N.A 1.08 (0.44; 2.65)
HA of 100% fruit juice Never/Decreased Improved Often	Ref. 0.90 (0.58; 1.11) 0.78 (0.60; 1.10)	Ref. 0.99 (0.64; 1.52) 0.85 (0.60; 1.20)	Ref. 0.86 (0.50; 1.47) 0.82 (0.54; 1.27)	Ref. 0.75 (0.26; 2.18) 0.53 (0.22; 1.29)
HA of vegetables Never/Decreased Improved Often	Ref. 1.88 (0.70; 5.03) 1.36 (0.73; 2.54)	Ref. 0.68 (0.18; 2.56) 0.61 (0.25; 1.47)	Ref. 3.32 (0.84; 13.18) 2.62 (0.88; 7.81)	Ref. 6.62 (0.55; 79.99) 6.03 (0.68; 53.74)
Modeling of fruit Never/Decreased Improved Often	Ref. 1.29 (0.89; 1.87) 1.30 (0.99; 1.70)	Ref. 1.22 (0.73; 2.05) 0.88 (0.61; 1.26)	Ref. 0.79 (0.42; 1.49) 1.15 (0.73; 1.83)	Ref. 0.78 (0.25; 2.39) 0.83 (0.32; 2.20)
**Negative Food Parenting Practices**				
HA of sugar juices Often/Increased Improved (decreased) Never	Ref. **2.81 (2.09; 3.79)** **5.35 (4.00; 7.18)**	Ref. 1.08 (0.74; 1.57) 1.33 (0.91; 1.93)	Ref. 1.68 (1.00; 2.82) **1.92 (1.19; 3.10)**	Ref. 1.50 (0.51; 4.44) **3.63 (1.32; 10.00)**
HA of soft drinks Often/Increased Improved (decreased) Never	Ref. **3.14 (2.20; 4.49)** **6.14 (4.53; 8.30)**	Ref. **0.60 (0.40; 0.92)** 1.12 (0.77; 1.63)	Ref. **1.94 (1.06; 3.55)** **2.15 (1.30; 3.57)**	Ref. 1.09 (0.30; 3.90) **3.88 (1.45; 10.36)**
HA of light soft drinks Often/Increased Improved (decreased) Never	Ref. 1.41 (0.95; 2.10) **1.44 (1.09; 1.90)**	Ref. **5.11 (2.83; 9.24)** **12.59 (8.06; 19.69)**	Ref. 1.19 (0.61; 2.32) **1.86 (1.16; 2.98)**	Ref. 0.31 (0.03; 3.59) **3.48 (1.32; 9.17)**
HA of sweets Often/Increased Improved (decreased) Never	Ref. **1.45 (1.06; 1.96)** 2.18 (0.86; 5.54)	Ref. 0.93 (0.61; 1.41) 0.60 (0.21; 1.75)	Ref. **2.36 (1.52; 3.67)** **10.98 (4.41;27.36)**	Ref. 1.14 (0.41; 3.16) 1.56 (0.10; 24.42)
HA of salty snacks Often/Increased Improved (decreased) Never	Ref. 1.15 (0.84; 1.57) **1.85 (1.35; 2.54)**	Ref. 0.92 (0.60; 1.40) 1.06 (0.69; 1.64)	Ref. **2.55 (1.52; 4.28)** **3.65 (2.22; 5.98)**	Ref. 1.88 (0.57; 6.15) **9.31 (3.28; 26.44)**
Allowance of salty snacks Often/Increased Improved (decreased) Never	Ref. 1.23 (0.91; 1.66) **1.63 (1.21; 2.18)**	Ref. 0.83 (0.56; 1.11) 1.06 (00.72; 1.57)	Ref. **1.87 (1.14; 3.06)** **2.83 (1.78; 4.48)**	Ref. **2.97 (1.12; 7.84)** **8.83 (3.00; 25.96)**
Use of food as a reward Often/Increased Improved (decreased) Never	Ref. 0.97 (0.65; 1.47) **1.83 (1.36; 2.47)**	Ref. 1.19 (0.70; 2.04) 1.25 (0.85; 1.84)	Ref. 1.16 (0.53; 2.55) **1.76 (1.00; 3.09)**	Ref. 1.78 (0.22; 6.36) 2.96 (0.86; 10.15)

N = 1494 *, except for salty snacks ^†^, *n* = 1268. Abbreviations: OR, odds ratio (odds for being allocated in the group that follows recommendations); 95% CI, 95% confidence interval; HA, home availability; T0, baseline period, T1, follow-up period, CI, confidence intervals; Ref, reference category. Multilevel logistic regression was adjusted for BMI at T0 and T1, sex, age, parental education, and center. Categories of FPP indicate a change in the use of them over time. All models of the multilevel logistic regression include random effects (country and group) to account for the study design. Ref. for energy-dense/nutrient-poor foods: one serving or less per week for each food item.

**Table 7:** Multilevel logistic regression analysis by combinations of change for food parenting practices over time (T0 to Table 2. and its effects on the compliance of recommendations for energy-dense/nutrient-poor foods at follow-up in boys, *N =* 1473 *.

	Categories of Energy-Dense/Nutrient-Poor Foods ^†^			
	Soft Drinks and Sugar Juices	Light Soft Drinks	Sweets	Salty Snacks and Fast Foods
	OR (95% CI)	OR (95% CI)	OR (95% CI)	OR (95% CI)
**Positive Food Parenting Practices**				
HA of fruit Never/Decreased Improved (increased) Often	Ref. **0.37 (0.15; 0.93)** 0.79 (0.41; 1.50)	Ref. 1.19 (0.30; 4.62) 0.74 (0.31; 1.79)	Ref. 0.43 (0.10; 1.76) 0.49 (0.20; 1.20)	Ref. 0.62 (0.21; 1.82) 1.11 (0.54; 2.26)
HA of 100% fruit juice Never/Decreased Improved Often	Ref. 1.19 (0.87; 1.65) 1.20 (0.92; 1.55)	Ref. 0.82 (0.55; 1.22) 0.92 (0.66; 1.30)	Ref. 1.09 (0.59; 2.02) 1.24 (0.76; 2.00)	Ref. 0.96 (0.68; 1.37) 1.06 (0.80; 1.39)
HA of vegetables Never/Decreased Improved Often	Ref. **0.35 (0.12; 0.99)** 0.77 (0.37; 1.59)	Ref. 0.68 (0.15; 3.15) 0.70 (0.24; 2.10)	Ref. 0.91 (0.14; 5.68) 1.55 (0.40; 6.05)	Ref. 1.18 (0.36; 3.84) 2.21 (0.93; 5.27)
Modeling of fruit Never/Decreased Improved Often	Ref. 1.00 (0.985; 1.46) 1.14 (0.87; 1.49)	Ref. 0.92 (0.56; 1.51) 0.78 (0.55; 1.11)	Ref. 0.83 (0.42; 1.62) 0.86 (0.54; 1.37)	Ref. 0.92 (0.61; 1.37) **1.33 (1.00; 1.75)**
**Negative Food Parenting Practices**				
HA of sugar juices Often/Increased Improved (decreased) Never	Ref. **2.06 (1.55; 2.74)** **5.48 (4.09; 7.35)**	Ref. 1.05 (0.74; 1.50) **1.73 (1.19; 2.51)**	Ref. 1.06 (0.61; 1.86) **1.80 (1.11; 2.91)**	Ref. 1.33 (0.98; 1.82) **1.76 (1.33; 2.34)**
HA of soft drinks Often/Increased Improved (decreased) Never	Ref. **2.45 (1.74; 3.43)** **5.13 (3.84; 6.86)**	Ref. 0.78 (0.52; 1.16) 1.42 (0.99; 2.04)	Ref. **2.31 (1.21; 4.44)** **2.27 (1.29; 3.99)**	Ref. **1.76 (1.21; 2.56)** **2.23 (1.66; 3.01)**
HA of light soft drinks Often/Increased Improved (decreased) Never	Ref. 1.19 (0.80; 1.76) **1.53 (1.16; 2.01)**	Ref. **2.36 (1.44; 3.88)** **9.02 (5.99; 13.58)**	Ref. **2.07 (1.07; 3.98)** **1.75 (1.03; 2.96)**	Ref. **1.66 (1.08; 2.55)** **1.49 (1.11; 2.00)**
HA of sweets Often/Increased Improved (decreased) Never	Ref. 1.20 (0.88; 1.64) 1.93 (0.87; 4.28)	Ref. 0.87 (0.57; 1.31) 1.27 (0.43; 3.74)	Ref. **2.96 (1.84; 4.78)** **18.55 (7.35;46.79)**	Ref. **1.74 (1.26; 2.40)** **4.16 (1.84; 9.39)**
HA of salty snacks Often/Increased Improved (decreased) Never	Ref. 1.16 (0.86; 1.57) **2.57 (1.85; 3.59)**	Ref. 1.18 (0.79; 1.77) **2.12 (1.29; 3.49)**	Ref. **2.51 (1.46; 4.32)** **3.57 (2.09; 6.10)**	Ref. **2.39 (1.71; 3.33)** **5.36 (3.58; 7.46)**
Allowance of salty snacks Often/Increased Improved (decreased) Never	Ref. 1.12 (0.83; 1.51) **1.39 (1.03; 1.89)**	Ref. 0.90 (0.61; 1.34) 1.00 (0.68; 1.46)	Ref. 0.64 (0.36; 1.16) 1.49 (0.89; 2.50)	Ref. 0.98 (0.71; 1.35) 1.14 (0.83; 1.56)
Use of food as a reward Often/Increased Improved (decreased) Never	Ref. 1.13 (0.77; 1.65) **1.59 (1.18; 2.14)**	Ref. 0.88 (0.56; 1.40) 1.25 (0.86; 1.82)	Ref. 1.16 (0.39; 3.41) **4.20 (1.95; 9.07)**	Ref. **2.42 (1.58; 3.70)** **2.30 (1.63; 3.25)**

N= 1473 *, except for salty snacks ^†^, *n* = 1232. Abbreviations: OR, odds ratio (odds for being allocated in the group that follows recommendations); 95% CI, 95% confidence interval; HA, home availability; T0, baseline period, T1, follow-up period, CI, confidence intervals; Ref, reference category. Multilevel logistic regression was adjusted for BMI at, T0 and T1, sex, age, parental education, and center. Categories of FPP indicate a change in the use of them over time. All models of the multilevel logistic regression include random effects (country and group) to account for the study design. Ref. for energy-dense/nutrient-poor foods: one serving or less per week for each food item. (*) Unhealthy foods such as sweets, salty snacks, and fast food.

## Data Availability

The datasets generated and/or analyzed during the current study are not publicly available, since the data used is confidential based on Feel4Diabetes publications rules but are available from the corresponding author on reasonable request.

## Supplementary Materials

The following are available online at https://www.mdpi.com/article/10.3390/nu13041298/s1, Table S1. Questions, response options, and analytic coding for the analyses. The Feel4Diabetes study, Table S2. Compliance with recommendations for dietary intake in children from the Feel4Diabetes study at follow-up, *n* = 2967 *, Table S3. Cross-sectional associations between food parenting practices and dietary intake in girls in servings/day at baseline. Results from adjusted individual linear regressions, Table S4. Cross-sectional associations between food parenting practices and dietary intake in boys in servings/day at baseline. Results from adjusted individual linear regressions.
