# Supplementary material for: Longitudinal Associations between Food Parenting Practices and Dietary Intake in Children: The Feel4Diabetes Study
Source: Nutrients. 2021 Apr 14;13(4):1298. doi: 10.3390/nu13041298 (PMC8071003; doi:10.3390/nu13041298)
Supplement: Supplementary file 1 [file nutrients-13-01298-s001.pdf]

Supplementary Files

Table S1. Questions, response options, and analytic coding for the analyses. The Feel4Diabetes-study

Questionnaire item	Response options	Analytic coding
**Children's dietary intake**		
**Please indicate how often you and your child regularly consume the following foods and drinks.**		
1. Water (1 glass or 1 cup)<br>2. Fruits and berries, fresh or frozen (1 tennis ball-sized fruit, or 2 small fruits, or half a cup of chopped)<br>3. Fruits and berries, canned (half a cup) or dried (a quarter of a cup)<br>4. Fruit juices, freshly squeezed or prepacked without sugar (1 glass or 1 cup)<br>5. Soft drinks and juices containing sugar (1 glass or 1 cup)<br>6. Soft drinks, diet (light) (1 glass or 1 cup)<br>7. Vegetables (half a cup or the size of a tennis ball of tomato, broccoli, leafy vegetables, etc.)<br>8. Sweets (1 chocolate bar or half a cup of sweets, cookies, or ice cream)<br>9. Salty snacks/fast food (e.g. 1 small hamburger, 1 small bag of chips, 1 slice of pizza)	8 response options:<br>a. Less than once per week<br>b. 1–2 times per week<br>c. 3–4 times per week<br>d. 5–6 times per week<br>e. 1–2 times per day<br>f. 3–4 times per day<br>g. 5–6 times per day<br>h. More than 6 times per day	A continuous variable indicating servings per day was created according to the frequency of intake<br>a. Less than 1 t/w = 0.14 s/d<br>b. 1–2 times per week = 0.21 s/d<br>c. 3–4 t/w = 0.5 s/d<br>d. 5–6 t/w = 0.79 s/d<br>e. 1–2 t/d = 1.5 s/d<br>f. 3–4 t/d = 3.5 s/d<br>g. 5–6 t/d = 5.5 s/d<br>h. >6 t/d = 6 s/d
**Food parenting practices**		
**On a weekly basis, how frequently are the following food items available in your home?**		
1. Fruits<br>2. Fruit juices, freshly-squeezed or prepacked without sugar<br>3. Fruit juices, prepacked, containing sugar<br>4. Sugar juices<br>5. Soft drinks containing sugar<br>6. Soft drinks without sugar<br>7. Vegetables<br>8. Sweets, biscuits, ice cream, cakes, pastries<br>9. Salty snacks (e.g. chips, savoury pastries)	5 response options:<br>a. Always<br>b. Often<br>c. Sometimes<br>d. Rarely<br>e. Never	Categories were recorded from never to always and afterwards recategorized into three groups:<br>1=Never/rarely<br>2= Sometimes<br>3=Often/always
**On a weekly basis, how often do you use the following practices:**		
10. How often do you consume fresh fruits with your child?<br>11. How often do you reward your child with sweets, salty snacks (e.g. potato chips), or fast food?<br>12. How often do you allow your child to eat sweets and/or salty snacks whenever he/she asks for them?	a. Very often<br>b. Often<br>c. Sometimes<br>d. Rarely<br>e. Never	Categories were recorded from never to very often and afterwards recategorized into three groups:<br>1=Never/rarely<br>2= Sometimes<br>3=Often/Very often

Bold text is as used in the questionnaire.

Table S2. Compliance with recommendations for dietary intake in children from the Feel4Diabetes-study at follow-up, n = 2967*

	Total		Girls		Boys	
**Nutrient-dense foods**	**No**	**Yes**	**No**	**Yes**	**No**	**Yes**
Water	84.0 (2491)	16.0 (476)	84.7 (1265)	15.3 (229)	83.2 (1226)	16.8 (247)
Fruits & berries	48.4 (1435)	51.6 (1532)	48.5 (725)	51.5 (769)	48.2 (710)	51.8 (763)
Canned fruits	97.9 (2905)	2.1 (62)	97.7 (1460)	2.3 (34)	98.1 (1445)	1.9 (28)
100% fruit juice	87.0 (2582)	13.0 (385)	88.7 (1325)	11.3 (169)	85.3 (1257)	14.7 (216)
Vegetables	52.8 (1568)	47.2 (1399)	53.1 (793)	46.9 (701)	52.6 (775)	47.4 (698)
F&V[1]	92.7 (2749)	7.3 (218)	92.8 (1387)	7.2 (107)	92.5 (1362)	7.5 (111)
F&V[2]	91.6 (2717)	8.4 (250)	92.0 (1374)	8.0 (120)	91.2 (1343)	8.8 (130)
**Energy-dense/nutrient-poor foods**						
Soft drinks & sugar juices	44.2 (1310)	55.8 (1657)	41.8 (625)	58.2 (869)	46.5 (685)	53.5 (788)
Light soft drinks	19.3 (574)	80.7 (2393)	18.6 (278)	81.4 (1216)	20.1 (296)	79.9 (1177)
Sweets	90.9 (2698)	9.1 (269)	89.8 (1341)	10.2 (153)	92.1 (1357)	7.9 (116)
Salty snacks & fast food[+]	62.3 (1557)	37.7 (943)	60.6 (768)	39.4 (500)	64.0 (789)	36.0 (443)

* N= 2967, except salty snacks N= 2500. Recommendations were based on the Irish Food Pyramid. F&V[1]: indicates the sum of servings of fresh or frozen fruit & berries, fresh fruit juices, and vegetables. F&V[2]: Same as in Fruits & vegetables[1] + canned fruits & berries.

Cut-off points for recommendations for healthy foods: water = 6 or more servings per day; fruits & berries, canned fruit, fresh fruit juice & vegetables = 1–2 servings per day, F&V[1] and F&V[2]= 5 or more servings per day and for unhealthy foods: 1 serving or less per week for each food item. ([+]) Unhealthy foods like sweets, salty snacks, and fast food.

Table S3. Cross-sectional associations between food parenting practices and dietary intake in girls in servings/day at baseline.

	**Nutrient-dense foods**							**Energy-dense/nutrient-poor foods**			
	**Water**	**Fruits & berries**	**Canned fruits & berries**	**Fresh fruit juice**	**Vegetables**	**F&V**[1]	**F&V**[2]	**Soft drinks & sugar juices**	**Light soft drinks**	**Sweets**	**Salty snacks & fast food**
	β (p-value)	β (p-value)	β (p-value)	β (p-value)	β (p-value)	β (p-value)	β (p-value)	β (p-value)	β (p-value)	β (p-value)	β (p-value)
**Positive FPP**											
HA Fruit	0.007 (0.759)	**0.282 (<0.001)**	0.015 (0.552)	0.048 (0.052)	**0.169 (<0.001)**	**0.263 (<0.001)**	**0.257 (<0.001)**	**-0.075 (0.004)**	**-0.060 (0.020)**	-0.012 (0.619)	**-0.068 (0.012)**
HA 100% fruit juice	0.003 (0.891)	**0.069 (0.013)**	**0.088 (0.001)**	**0.492 (<0.001)**	**0.059 (0.021)**	**0.217 (<0.001)**	**0.222 (<0.001)**	0.024 (0.386)	0.029 (0.281)	**-0.054 (0.032)**	-0.007 (0.815)
HA Vegetables	0.038 (0.117)	**0.229 (<0.001)**	-0.013 (0.629)	**0.028 (0.271)**	**0.313 (<0.001)**	**0.299 (<0.001)**	**0.287 (<0.001)**	-0.016 (0.560)	0.011 (0.680)	0.042 (0.087)	-0.044 (0.119)
Modelling of fruit intake	**0.109 (<0.001)**	**0.431 (<0.001)**	**0.108 (<0.001)**	**0.087 (<0.001)**	**0.269 (<0.001)**	**0.418 (<0.001)**	**0.421 (<0.001)**	**-0.059 (0.022)**	-0.003 (0.908)	**-0.078 (0.001)**	**-0.066 (0.014)**
**Negative FPP**											
HA Sugar juices	-0.045 (0.059)	**-0.077 (0.004)**	0.040 (0.124)	**0.072 (0.004)**	**-0.064 (0.009)**	-0.048 (0.070)	-0.044 (0.100)	**0.393 (<0.001)**	0.030 (0.263)	**0.113 (<0.001)**	**0.141 (<0.001)**
HA Soft drinks	**-0.082 (0.002)**	**-0.061 (0.042)**	-0.008 (0.788)	0.003 (0.917)	**-0.106 (<0.001)**	**-0.088 (0.003)**	**-0.084 (0.005)**	**0.367 (<0.001)**	0.015 (0.605)	**0.096 (<0.001)**	**0.152 (<0.001)**
HA Light soft drinks	0.012 (0.645)	0.010 (0.733)	0.011 (0.697)	0.036 (0.174)	-0.040 (0.130)	-0.003 (0.929)	0.000 (0.996)	0.032 (0.256)	**0.255 (<0.001)**	0.047 (0.073)	**0.084 (0.005)**
HA Sweets	-0.047 (0.070)	**-0.100 (0.001)**	**-0.075 (0.009)**	**-0.082 (0.002)**	**-0.080 (0.003)**	**-0.124 (<0.001)**	**-0.129 (<0.001)**	**0.106 (<0.001)**	0.040 (0.159)	**0.393 (<0.001)**	**0.197 (<0.001)**
HA Salty snacks	-0.008 (0.768)	**-0.099 (0.001)**	-0.054 (0.068)	-0.009 (0.749)	**-0.092 (0.001)**	**-0.105 (<0.001)**	**-0.109 (<0.001)**	**0.110 (<0.001)**	0.003 (0.923)	**0.228 (<0.001)**	**0.381 (<0.001)**
Permissiveness	**-0.061 (0.010)**	**-0.146 (<0.001)**	-0.049 (0.061)	0.032 (0.193)	**-0.117 (<0.001)**	**-0.126 (<0.001)**	**-0.127 (<0.001)**	**0.178 (<0.001)**	0.016 (0.543)	**0.275 (<0.001)**	**0.255 (<0.001)**
Use of foods as reward*	**-0.083 (<0.001)**	**-0.060 (0.023)**	0.024 (0.348)	0.025 (0.317)	**-0.099 (<0.001)**	**-0.086 (0.001)**	**-0.081 (0.002)**	**0.144 (<0.001)**	0.023 (0.366)	**0.151 (<0.001)**	**0.170 (<0.001)**

N= 1494, except salty snacks N = 1268. Linear regressions were performed individually (one for each practice) and were adjusted for country, group (control vs. intervention), parental education, sex, age, and BMI and children's gender, age, and z-BMI. β = Standardized coefficients; FPP, food parenting practices; HA, home availability. Boldface indicates statistical significance at $p \leq 0.05$. (*) Energy-dense/nutrient-poor foods like sweets, salty snacks, and fast food.

Table S4. Cross-sectional associations between food parenting practices and dietary intake in boys in servings/day at baseline.

	Nutrient-dense foods							Energy-dense/nutrient-poor foods			
	**Water** β (p-value)	**Fruits & berries** β (p-value)	**Canned fruits & berries** β (p-value)	**Fresh fruit juice** β (p-value)	**Vegetables** β (p-value)	**F&V[1]** β (p-value)	**F&V[2]** β (p-value)	**Soft drinks & sugar juices** β (p-value)	**Light soft drinks** β (p-value)	**Sweets** β (p-value)	**Salty snacks & fast food** β (p-value)
**Positive FPP**											
HA Fruit	0.033 (0.163)	**0.321 (<0.001)**	0.022 (0.409)	**0.085 (0.001)**	**0.216 (<0.001)**	**0.322 (<0.001)**	**0.317 (<0.001)**	**-0.068 (0.010)**	0.025 (0.349)	0.002 (0.926)	**-0.119 (<0.001)**
HA 100% fruit juice	-0.040 (0.106)	**0.106 (<0.001)**	**0.059 (0.029)**	**0.506 (<0.001)**	**0.093 (<0.001)**	**0.265 (<0.001)**	**0.270 (<0.001)**	**-0.096 (<0.001)**	**0.079 (0.004)**	-0.031 (0.218)	-0.023 (0.430)
HA Vegetables	0.022 (0.364)	**0.208 (<0.001)**	0.031 (0.242)	**0.078 (0.002)**	**0.319 (<0.001)**	**0.309 (<0.001)**	**0.306 (<0.001)**	0.023 (0.378)	0.004 (0.866)	-0.003 (0.887)	**-0.069 (0.015)**
Modelling of fruit intake	**0.059 (0.011)**	**0.448 (<0.001)**	**0.056 (0.027)**	**0.106 (<0.001)**	**0.234 (<0.001)**	**0.394 (<0.001)**	**0.393 (<0.001)**	**-0.093 (<0.001)**	0.001 (0.959)	**-0.051 (0.032)**	**-0.088 (0.001)**
**Negative FPP**											
HA Sugar juices	**-0.090 (<0.001)**	**-0.060 (0.021)**	**0.057 (0.024)**	**0.079 (0.001)**	**-0.061 (0.013)**	-0.028 (0.279)	-0.023 (0.384)	**0.432 (<0.001)**	**0.051 (0.046)**	**0.120 (<0.001)**	**0.135 (<0.001)**
HA Soft drinks	**-0.069 (0.007)**	**-0.159 (<0.001)**	0.016 (0.565)	0.026 (0.337)	**-0.107 (<0.001)**	**-0.129 (<0.001)**	**-0.122 (<0.001)**	**0.319 (<0.001)**	0.024 (0.388)	**0.126 (<0.001)**	**0.186 (<0.001)**
HA Light soft drinks	**-0.083 (0.001)**	-0.040 (0.160)	-0.017 (0.536)	**0.074 (0.006)**	**-0.056 (0.038)**	-0.015 (0.592)	-0.015 (0.601)	0.034 (0.233)	**0.208 (<0.001)**	0.026 (0.322)	0.037 (0.230)
HA Sweets	**-0.053 (0.038)**	**-0.095 (0.001)**	**-0.096 (0.001)**	-0.016 (0.556)	**-0.060 (0.027)**	**-0.082 (0.004)**	**-0.092 (0.001)**	**0.149 (<0.001)**	-0.023 (0.419)	**0.393 (<0.001)**	**0.195 (<0.001)**
HA Salty snacks	**-0.073 (0.005)**	**-0.101 (0.001)**	**-0.073 (0.010)**	0.009 (0.758)	**-0.084 (0.002)**	**-0.084 (0.004)**	**-0.090 (0.002)**	**0.201 (<0.001)**	-0.22 (0.445)	**0.184 (<0.001)**	**0.410 (<0.001)**
Permissiveness	**-0.072 (0.003)**	**-0.128 (<0.001)**	-0.050 (0.055)	-0.010 (0.691)	**-0.099 (<0.001)**	**-0.111 (<0.001)**	**-0.117 (<0.001)**	**0.170 (<0.001)**	**0.070 (0.009)**	**0.261 (<0.001)**	**0.250 (<0.001)**
Use of foods as reward*	-0.026 (0.260)	**-0.032 (0.222)**	0.019 (0.467)	-0.005 (0.854)	**-0.012 (0.630)**	-0.004 (0.886)	-0.003 (0.904)	**0.085 (0.001)**	-0.005 (0.848)	**0.145 (<0.001)**	**0.150 (<0.001)**

N= 1473, except salty snacks N= 1232. Linear regressions were performed individually (one for each practice) and were adjusted for country, group (control vs. intervention), parental education, sex, age, and BMI and children's gender, age, and z-BMI. β = Standardized coefficients; FPP, food parenting practices; HA, home availability. Boldface indicates statistical significance at $p \leq 0.05$. (*) Energy-dense/nutrient-poor foods like sweets, salty snacks, and fast food.
